# Retrospective evaluation of tooth injuries and associated factors at a hospital emergency ward

**DOI:** 10.1186/s12903-015-0125-4

**Published:** 2015-11-04

**Authors:** Meire Coelho Ferreira, Anne Margareth Batista, Leandro Silva Marques, Fernanda de Oliveira Ferreira, João Batista Medeiros-Filho, Maria Letícia Ramos-Jorge

**Affiliations:** Dentistry Department, Ceuma University, R Perdizes, 27/ 805, Quadra 35, Edifício University Home, São Luís, MA 65.075-340 Brazil; Dentistry Department, School of Biological and Health Sciences, Federal University of the Jequitinhonha and Mucuri Valleys, Diamantina, Minas Gerais Brazil; Department of Basic Sciences, Federal University of Juiz de Fora, Governador Valadares, Minas Gerais Brazil

**Keywords:** Tooth injuries, Soft tissues injuries, Risk factors

## Abstract

**Background:**

The aim of study was to determine the occurrence of tooth injuries and associated factors among patients treated at a hospital emergency ward.

**Methods:**

A cross-sectional study was conducted involving the analysis of 790 patient charts. The independent variables were gender, place of residence and type of accident. The dependent variable was tooth injury (fractures, concussion, luxation and avulsion). Statistical analysis involved the chi-square test, Poisson analysis and logistic regression. Explanatory variables with a p-value < 0.20 in the bivariate analysis were incorporated into the multivariate model.

**Results:**

A total of 681 (86.2 %) patients had tooth injury, among whom 159 (20.1 %) had tooth fractures. Tooth concussion was associated with residence in urban areas (PR = 1.635; 95 % CI: 1.199-2.230), the male gender (PR = 1.673; 95 % CI: 1.225-2.285), violence (PR = 1.940; 95 % CI: 1.263-2.982) and sports (PR = 1.863; 95 % CI: 1.287-2.696). The prevalence rate of tooth fracture was higher among individuals having suffered a motorcycle (PR = 1.597; 95 % CI: 1.295-1.968) or bicycle accident (PR = 1.484; 95 % CI: 1.245-1.769). Victims of bicycle accidents had a 42.6-fold greater chance of suffering luxation (95 % CI: 20.917-86.808) and a threefold greater chance of suffering avulsion (95 % CI: 1.620-5.848). Victims of motorcycle accidents had a 2.96-fold greater chance of suffering avulsion (95 % CI: 1.471-5.937).

**Conclusions:**

In the study, concussion was the most frequent type of tooth injury. Motorcycle and bicycle accidents were associated with tooth fractures, luxation and avulsion, whereas sports and violence were associated with dental concussion. The findings on tooth injuries can contribute to public health policies regarding the prevention and health promotion measures.

## Background

The increase in violence, traffic accidents and participation in sport activities has contributed to the occurrence of tooth injuries (TIs), which are considered a public health problem [1, 2] and a challenge to dentists [3]. Maxillofacial and dental injuries accounts for 5 % of all injuries-related hospitalizations [4]. The treatment of such cases is often complex and expensive, requiring the participation of specialists from different fields.

Most TIs affect the anterior teeth, especially the maxillary central and lateral incisors [5–7], which can have physical, esthetic and psychological impacts [8, 9]. Enamel fracture is the most common type of tooth injury (TI) [9–12]. Retrospective data report traffic accidents and falls as the most frequent etiological factors of TI [4, 7, 10, 13].

The identification of groups at risk, the evaluation of demands for treatment regarding TI at healthcare services and the development of prevention and clinical protocols for individuals affected by TI depend directly on knowledge regarding the specific situation of each community. Thus, the evaluation of TI is necessary to improving the quality of evidence on this subject.

The aim of the present study was to determine the occurrence of tooth injuries and associated factors among patients treated at a hospital emergency ward.

## Methods

This study received approval from the research ethics committee of the *Imaculada Conceição* Regional Hospital (Health Centers National Register: 2144530). All patients sign a statement of informed consent when are admitted to hospital, authorizing the use of clinical and radiographic findings for research purposes. A cross-sectional study was conducted involving the analysis of 1121 charts of patients treated at the emergency ward of the *Imaculada Conceição* Regional Hospital in the municipality of Guanhães (Brazil) between January 2005 and December 2007. This hospital is a reference center for the urban and rural areas of 23 municipalities of the region denominated *Vale do Rio Doce* in the state of Minas Gerais (Brazil), reaching a total population of approximately 238,797 inhabitants in an area spanning 12,745 km^2^. The inclusion criterion was a complete record of oral-maxillofacial trauma. Among the 1121 charts analyzed, 790 charts met this criterion. The evaluation of the charts was performed for an examiner specialist in oral and maxillofacial surgery, responsible for the diagnosis and treatment of patients with oral-maxillofacial and dental injuries.

The sample size calculation was carried out considering the study design. The study presented an exploratory objective (to determine the occurrence of tooth injuries) and an analytical aim (to determine whether gender, age and type of accident are factors associated with tooth injuries). To determine the occurrence of tooth injuries, it was necessary to calculate the sample size appropriated. The sample size was estimated based on a 50 % occurrence rate of tooth injuries, a 4 % level of precision, and 95 % confidence interval (CI). The minimum sample size was estimated at 600 patients.

Considering the analytical aim, we performed the sample size calculation for the analytical cross-sectional study, considering the type I and type II errors. It was carried out the sample size calculation to compare proportions between groups (tooth injuries’s occurrence between gender, age and type of accidents). It was considered P = 50 %, as it was unknown the tooth injuries’s occurrence in this region. The calculation was performed adopting 95 % significance level, 80 % of statistical power, and assuming a minimum difference to be detected of 10 % of trauma occurrence between gender, age and type of accidents, resulting in a maximum sample size of 784 individuals. Thus, 790 individuals were considered appropriate for the exploratory and analytical aims.

The following information was extracted from the patient charts: socio-demographic data, TI (tooth fractures, concussion, luxation and avulsion) [14], soft tissue injuries and type of accident.

Descriptive statistics were performed for the socio-demographic data, TI and type of accident. Unadjusted and adjusted regression analyses were performed to investigate explicative factors of TI. To avoid overestimating the odds ratio (OR), Poisson analysis was performed when the occurrence of the type of TI was higher than 10 % in the population studied. Logistic regression was performed when the occurrence of the outcome was less than 10 %. Independent variables with a p-value < 0.20 in the unadjusted analysis were incorporated into the adjusted regression model. The p value for keeping variables in the adjusted model was set at 0.05. The Statistical Package for Social Sciences (SPSS for Windows, version 15.0, SPSS Inc. Chicago, IL, USA) was used for the analyses.

## Results

Table 1 displays the socio-demographic data and types of accident. Dental concussion was the most prevalent type of TI (78 %). Tables 2, 3 and 4 display the occurrence of tooth fractures and periodontal tissue injury according to gender, age and type of accident. The type of accident according to age is presented in the Table 5.

**Table 1:** Distribution of socio-demographic variables and types of accident. Guanhães, Brazil, 2005-2007 (n = 790)

Variable	n (%)
Gender	
Male	537 (68.0)
Female	253 (32.0)
Age^a^	
<13 years	83 (10.5)
13 to 19 years	205 (25.9)
20 to 29 years	317 (40.1)
≥30 years	185 (23.4)
Residence	
Urban areas	534 (67.6)
Rural areas	256 (32.4)
Type of accident	
Automobile accident	59 (7.5)
Motorcycle accident	72 (9.1)
Bicycle accident	87 (11.0)
Accident with animal	111 (14.1)
Work-related accident	40 (5.1)
Violence	150 (19.0)
Sports	162 (20.5)
Fall	115 (14.6)

^a^Mean age: 25.75 ± 14.11 years

**Table 2:** Distribution of tooth injuries according to gender. Guanhães, Brazil, 2005-2007 (n = 790)

Type of tooth injury	Male	Female	Total (%)
n (%)	n (%)	n (%)	
Fractures	116 (73.0)	43 (27.0)	159 (20.1)
Concussion	404 (65.6)	212 (34.4)	616 (78.0)
Luxation	31 (63.3)	18 (36.7)	49 (6.2)
Avulsion	44 (65.7)	23(34.3)	67 (8.5)

Fractures = Enamel fracture, enamel + dentin fracture and enamel + dentin fracture with pulp involvement

**Table 3:** Distribution of tooth injuries according to age group. Guanhães, Brazil, 2005-2007 (n = 790)

Type of tooth injury	Age group				
	<13 years	13-19 years	20-29 years	≥30 years	Total (%)
	n (%)	n (%)	n (%)	n (%)	
Fractures	22 (13.8)	43 (27.0)	69 (43.4)	25 (15.7)	159 (20.1)
Concussion	63 (10.2)	167 (27.1)	251 (40.7)	135 (21.9)	616 (78.0)
Luxation	6 (12.2)	13 (26.5)	20 (40.8)	10 (20.4)	49 (6.2)
Avulsion	10 (14.9)	21 (31.3)	23 (34.3)	13 (19.4)	67 (8.5)

**Table 4:** Distribution of tooth injuries according to type of accident. Guanhães, Brazil, 2005-2007 (n = 790)

Type of tooth injury	Automobile accident	Motorcycle accident	Bicycle accident	Accident with animal	Work-related accident	Violence	Sports	Fall	Total (%)
	n (%)	n (%)	n (%)	n (%)	n (%)	n (%)	n (%)	n (%)	
Fractures	13 (8.2)	32 (20.1)	35 (22)	25 (15.7)	10 (6.3)	2 (1.3)	39 (24.5)	2 (1.3)	159 (20.1)
Concussion	41 (6.7)	55 (8.9)	58 (9.4)	95 (15.4)	21 (3.4)	129 (20.9)	136 (22.1)	85 (13.8)	616 (78.0)
Luxation	3 (6.1)	1 (2.0)	37 (75.5)	2 (4.1)	4 (8.2)	0 (.0)	1 (2.0)	3 (6.1)	49 (6.2)
Avulsion	8 (11.9)	12 (17.9)	15 (22.4)	15 (22.4)	2 (3.0)	0 (.0)	13 (19.4)	2 (3.0)	67 (8.5)

**Table 5:** Distribution of type of accident according to age group. Guanhães, Brazil, 2005-2007 (n = 790)

Type of accident	Age group				
	<13 years	13-19 years	20-29 years	≥30 years	Total (%)
	n (%)	n (%)	n (%)	n (%)	
Automobile	3 (5.1)	8 (13.6)	30 (50.8)	18 (30.5)	59 (7.5)
Motorcycle	4 (5.6)	15 (20.8)	45 (62.5)	8 (11.1)	72 (9.1)
Bicycle	12 (13.8)	23 (26.4)	30 (34.5)	22 (25.3)	87 (11.0)
Accident with animal	0 (0)	29 (26.1)	45 (40.5)	37 (33.3)	111 (14.1)
Work-related accident	0 (0)	9 (22.5)	15 (37.5)	16 (40.0)	40 (5.1)
Violence	4 (2.9)	34 (24.3)	68 (48.6)	34 (24.3)	140 (17.7)
Sports	27 (16.7)	70 (43.2)	61 (37.7)	4 (2.5)	162 (20.5)
Falls	33 (28.7)	17 (14.8)	22 (19.1)	43 (37.4)	115 (14.6)

Residents of urban areas had a greater likelihood of suffering dental concussion than residents of rural areas. Moreover, the male gender had a greater likelihood of suffering dental concussion than the female gender. Violence and sports were associated with dental concussion. The occurrence rate of tooth fracture was higher among individuals having suffered a bicycle or motorcycle accident. Victims of bicycle accidents had a 42.6-fold greater chance of suffering luxation and a threefold greater chance of suffering avulsion. Victims of motorcycle accidents had a 2.96-fold greater chance of suffering avulsion (Table 6).

**Table 6:** Multiple regressions for tooth fractures and periodontal tissues injuries. Guanhães, Brazil, 2005-2007 (n = 790)

Univariate and multivariate Poisson regression for dental concussion and tooth fractures							
Dependent variables	Independent variables	Crude PR	95 % CI	*p*	Adjusted PR	95 % CI	*p*
Concussion	Residence^a^	1.416	1.041-1.927	0.027	1.635	1.199-2.230	0.002
	Gender^a^	1.528	1.114-2.098	0.009	1.673	1.225-2.285	0.001
	Violence	1.708	1.122-2.599	0.013	1.940	1.263-2.982	0.003
	Sports	1.468	1.005-2.146	0.047	1.863	1.287-2.696	0.001
Fractures	Motorcycle accident	1.482	1.202-1.827	0.000	1.597	1.295-1.968	0.000
	Bicycle accident	1.378	1.156-1.643	0.000	1.484	1.245-1.769	0.000
	Sports	1.065	0.969-1.171	0.190	1.168	1.065-1.281	0.001
Univariate and multivariate logistic regression for dental luxation and avulsion							
Dependent variables	Independent variables	Crude OR	95 % CI	p	Adjusted OR	95 % CI	p
Luxation	Bicycle accident	42.612	20.917-86.808	0.000	____	____	____
Avulsion	Motorcycle accident	2.411	1.224-4.750	0.011	2.955	1.471-5.937	0.002
	Bicycle accident	2.608	1.398-4.867	0.003	3.078	1.620-5.848	0.001

*PR* prevalence ratio, *OR* odds ratio, *CI* confidence interval

Fractures: Enamel fracture, enamel + dentin fracture and enamel + dentin fracture with pulp involvement grouped

^a^Reference categories: Residence – rural area; gender – female

## Discussion

In the present sample, dental concussion was the most frequent type of TI (78.0 %), followed by tooth fractures (20.1 %). All types of TI were more common in the male gender and the 20-to-29-year-old age group. The most prevalent etiological factors for tooth facture were motorcycle accidents, bicycle accidents and sports. Dental concussion was associated with violence and sports; luxation was associated with bicycle accidents; and avulsion was associated with bicycle accidents, accidents with animals, sports and motorcycle accidents. These findings are in agreement with data described in previous studies [4, 7, 10, 13].

Dental concussion is not the type of injury that leads people to seek dental treatment. In fact, most people have no knowledge of this problem and when the injury is isolated, it is not obviously visible. In cross-sectional studies involving samples from the general population, the majority of these injuries may have remained undiscovered, given that the time of assessment does not usually coincide with the occurrence of the injury, leading to underdiagnosis. Conversely, in studies involving samples from healthcare services, where data are evaluated retrospectively, this type of injury is usually diagnosed.

Although males were more affected by TI, the gender difference did not achieve statistical significance. This finding is in agreement with data described in a study conducted by Ajayi et al. [13], but is in disagreement with data described by Caldas Jr. and Burgos [10] and Guedes et al. [7]. According to the literature, the greater occurrence of TI in the male gender is due to a more active participation in hazardous work, sports and traffic [7, 15]. However, this has been changing, as the role of females increasingly resembles that of males in some societies [5].

Individuals aged 20 to 29 years of age were more affected by TI, with a predominance of injuries stemming from automobile accidents (50.8 %), motorcycle accidents (62.5 %), bicycle accidents (34.5 %), accidents with animals (40.5 %) and violence (48.6 %). This phase in life corresponds to more intensive social activity with regard to work and recreation, which leads to greater exposure to adverse situations. The present findings underscore the need for public policies and educational campaigns directed at reducing the rates of traffic accidents, violence, reckless behavior and accidents with animals, which can lead to bodily injuries and maxillofacial trauma.

The practice of sports accounted for the largest number of TIs (20.5 %), which may be explained by the fact that most of the sample resided in urban areas, where contact with sports is greater than in rural areas. In contrast, previous studies report a small frequency of TI stemming from sports [7, 10, 13]. In the study by Caldas Jr. and Burgos [9], the authors attribute the low prevalence rate of TI due to sports to the lower availability of recreational facilities in poor populations in the city of Recife, Brazil, since the emergency ward at which the study was conducted is located in an area with a low socioeconomic status. In the present study, the age of the sample (predominantly 13 to 29 years) may also explain why sports constituted the main etiological factor for TI, as this age group is known to practice sports more. Indeed, this was the age group most affected by TI stemming from sports (80.9 %). Moreover, sport-related TI accounted for 79.6 % of tooth injuries in the male gender.

Violence was the etiological factor of TI among 19 % of the present sample, with the male gender (59.3 %) and 20-to-29-year-old age group (48.6 %) the most affected. In a previously published study involving the same sample, violence was dichotomized as domestic and urban, with women more often victims of the former and men more often victims of the latter. The authors point out that the number of cases treated at the urgent and emergency care service over a two-year period did not correspond to the total number of cases of physical violence in the region, as underreporting occurs in cases of less severe trauma for which basic healthcare services and private clinics are capable of treating the injuries [2]. In other studies, the occurrence rate of TI stemming from violence ranges from 5.7 to 8.0 % [4, 7, 10]. In the study by Caldas Jr. and Burgos [10], TIs among women were related to violence as well as collisions with objects. The under-reporting of cases of physical violence could be associated with feelings of shame and fear experienced by the victim, who are usually female. A study was conducted to assess the pattern of oral and maxillofacial trauma caused by physical violence. Of the 42 cases involving domestic violence, 29 (69 %) involved female victims [2]. Recognizing the injury pattern caused by violence is important for the diagnosis of this type of case; the decision making process involved in the creation of health policy; assessments of the healthcare services required; and the development of prevention programs that aim to minimize the occurrence of violence.

Falls accounted for 14.6 % of TIs in the present sample and were more common among the female gender (51.3 %) and individuals aged 30 years or older (37.4 %). This finding is in disagreement with data reported by Caldas Jr. and Burgos [10] (72.4 %), Guedes et al. [7] (51.7 %), Levin et al. [4] (37.0 %) and Ajayi et al. [13] (34.0 %). The differences among studies may be the result of different sample sizes, sampling methods, diagnostic criteria, teeth evaluated (anterior teeth alone or all teeth) and behavioral patterns of the different populations studied. Therefore, comparisons should be made with caution. Individuals under the age of 13 years also exhibit an expressive frequency of falls (28.7 %). Elderly people tend to fall due to the lack of balance that comes with advancing age. For younger individuals, falls generally occur due to the greater amount of movement involved in playing games or sports.

Accidents with animals accounted for 14.1 % of TIs and occurred only among males (100 %) and predominantly in the 20-to-29-year-old age group (40.5 %), followed by individuals aged 30 years and older (33.3 %). According to a previous study [16], among the 111 individuals who suffered an accident with an animal, 78 resided in rural areas. As livestock farming is one of the main professional activities in the region where the data were collected, accidents with animals may be related to work, rodeos and means of transportation.

The use of secondary data is a limitation of the present study, as the precision and reliability of patient records are uncertain, but furnishes evidence of environmental factors of TI that can serve as reference for communities with similar characteristics to those of the sample analyzed.

## Conclusions

In the present study, concussion was the most frequent type of TI. Motorcycle and bicycle accidents were associated with tooth fractures, luxation and avulsion, whereas sports and violence were associated with dental concussion. The findings on TI can contribute to public health policies regarding the prevention and health promotion measures.

## Abbreviations

TIs: Tooth injuries
TI: Tooth injury
CI: Confidence interval
OR: Odds ratio.
